# Local Adaptation for Seasonal Cold Tolerance in a High‐Elevation Conifer Species, Subalpine Larch (*Larix lyallii* Parl.)

**DOI:** 10.1111/eva.70201

**Published:** 2026-02-19

**Authors:** Marie Vance, Barbara Hawkins, Jean Richardson, Patrick von Aderkas

**Affiliations:** ^1^ Department of Biology University of Victoria Victoria British Columbia Canada; ^2^ Ministry of Forests Victoria British Columbia Canada; ^3^ Environment & Climate Change Métis Nation of Ontario Ottawa Ontario Canada

**Keywords:** cold tolerance, electrolyte leakage, larix, subalpine larch

## Abstract

Subalpine larch ( *Larix lyallii* Parl.) is a deciduous conifer that only grows at treeline in the Cascade Range and Rocky Mountains of western North America. This habitat is shrinking due to climate change but subalpine larch is unlikely to migrate or adapt in situ and is therefore at risk of maladaptation and eventual extirpation. Future conservation efforts should be informed by an understanding of local adaptation in key traits. In this study, cold tolerance was assessed for 18 populations of subalpine larch from the Canadian portion of the species range that are grafted *ex situ* at the Kalamalka Forestry Centre in Vernon, BC. Electrolyte leakage was measured after stem tissue was subjected to artificial freezing tests at a range of subzero temperatures (−10°C to −40°C) over 2 years and three seasons (winter, spring, and autumn). Adaptive clines in cold tolerance were observed, providing the first evidence of local adaptation in this species. Temperature‐associated climate variables such as the length of the frost‐free period (FFP), the Julian date for the end of the frost‐free period, and mean coldest month temperature were significant predictors of cold injury at −40°C in all three seasons. Populations from colder sites with a shorter FFP were found to have significantly higher cold tolerance in all three seasons, with the biggest differences observed in spring and autumn. Future management strategies should prioritize the conservation of adaptive variation in cold tolerance.

## 1 Introduction

Populations of subalpine larch are threatened by climate change. This species grows within a restricted latitudinal range in the North Cascade Range and Rocky Mountains of western North America (Arno and Habeck 1972). A poor competitor in mixed stands, subalpine larch has carved out a niche at and above the altitudinal limits of other tree species in the transitional zone between the forest and alpine tundra ecosystems (1500–3000 m). Unfortunately, this habitat is disappearing. General circulation models predict warmer mean annual temperatures, earlier snowmelt and decreased summer precipitation in the mountains of western North America (Intergovernmental Panel on Climate Change 2013). Change is happening at a relatively rapid rate. By mid‐century, the alpine ecosystem in British Columbia, Canada, is projected to shrink by 64% (Wang et al. 2012). Because subalpine larch already grows on mountaintops, it has a limited ability to track its shifting climate niche via migration upward in elevation (Aitken et al. 2008). Furthermore, it is unlikely that subalpine larch will be able to adapt in situ to its changing environment. Demographic factors such as a long lifespan (average 500 years) and late arrival at sexual maturity (100–200 years; Arno and Habeck 1972) mean that any evolutionary response is likely to lag far behind a moving phenotypic optimum, leading to increasingly severe maladaptation and eventual extirpation (Lynch and Lande 1993). Active management will therefore be required to conserve populations of subalpine larch for future generations.

Conservation efforts should aim to preserve both overall genetic diversity and genetic diversity in ecologically important traits. A recent study of neutral genetic structure in subalpine larch identified three genetically differentiated regions across the species range: the Cascade Range, the southern Rocky Mountains and the northern Rocky Mountains (Vance et al. 2024). Differentiation likely arose after the last glacial maximum when populations expanded out of a central refugium on the Columbia Plateau (Roberts and Hamann 2015) but has since been reinforced by range disjunctions imposed by altitudinal retreats in response to Holocene climate warming. Management strategies based on neutral genetic markers typically seek to conserve the most genetically divergent groups and/or those groups with high levels of genetic diversity. Both criteria assume that neutral genetic variation reflects variation in ecologically important traits and that the retention of adequate standing genetic variation will ensure future adaptability. However, previous studies on neutral markers in conifers have found relatively low genetic differentiation over large geographic areas (Hamrick et al. 1992; Porth and El‐Kassaby 2014) even though genetic clines associated with climate gradients are ubiquitous (Alberto et al. 2013), suggesting that neutral markers frequently underestimate local adaptation in key traits. Assessing variation in ecologically important traits is therefore an important step for identifying populations that should be prioritized for management and conservation efforts.

Cold tolerance is a key trait for conifers in temperate and boreal forest ecosystems. Differences among species have long been understood to play a crucial role in defining range boundaries (Sakai and Weiser 1973), and phenotypic clines are frequently observed along latitudinal and altitudinal gradients (Aitken and Hannerz 2001; Alberto et al. 2013; MacLachlan et al. 2017). Local adaptation for cold tolerance allows populations to alternate between active growth and winter dormancy in synchrony with the annual climatic cycle. The growth/dormancy cycle involves a suite of traits, including the cessation of growth and bud set in late summer, cold acclimation in autumn, endodormancy and the attainment of maximum cold hardiness over winter, release from endodormancy, and deacclimation, the initiation of growth and budburst in spring. These traits are largely genetically independent and are triggered by different environmental cues. For example, the cessation of growth in summer occurs in response to decreasing photoperiod, although temperature, soil moisture, and plant nutritional status may also play a role. Release from endodormancy occurs after the accumulation of chilling units via prolonged exposure to non‐freezing low temperatures. Finally, the accumulation of heat sums triggers deacclimation, the initiation of growth and budburst. These traits have been shown to vary across species and populations. However, most studies on local adaptation in cold tolerance traits have focused on commercially important species with broad distributions at low elevation.

Subalpine larch thrives at treeline because of its extreme hardiness. This habitat has plentiful light, which is important for a deciduous species that must produce new needles every spring before achieving further carbon gains (Gower and Richards 1990), but it is a harsh environment. Across the species' range, the average temperature stays below freezing for at least half the year and the frost‐free period can last as few as 47 days. At the southern range margin, average monthly temperature only exceeds 10°C for two months of the year while at the northern range margin, average monthly temperature does not usually exceed 10°C. Despite these challenges, subalpine larch successfully balances its growth budget and avoids injury because it is well‐adapted to its environment. In the spring, subalpine larch begins to accumulate heat sums when air temperatures rise above 1.46°C (Worrall 1993) and initiates cone and needle growth when branches are no longer covered by snow, usually at the end of May (Arno and Habeck 1972). Decreasing photoperiod at the end of summer triggers growth cessation, leaf abscission, and bud dormancy. In winter, several adaptations are known to help subalpine larch cope with extreme cold. First, deciduousness is adaptive in that it removes the possibility of damage to leaves by snow and ice, as well as winter water loss through stomata and the cuticle (Berg and Chapin 1994). Winter desiccation is a serious problem for high‐elevation species that experience strong insolation while their roots remain frozen. Subalpine larch trees further avoid winter desiccation by isolating apical and lateral buds from xylem tissue (Richards and Bliss 1986). Finally, subalpine larch trees can tolerate extremely low water potential in their buds (Richards and Bliss 1986). This allows the buds to dehydrate over winter and avoid cellular freezing. These adaptations help subalpine larch thrive at treeline above the limits of other conifers.

In this study, local adaptation for seasonal cold tolerance was examined in 18 populations of subalpine larch grafted *ex situ* at the Kalamalka Forestry Centre in Vernon, BC. This clone bank is an invaluable resource because it is difficult to study phenology in natural populations of subalpine larch, which are inaccessible for most of the year. Cold injury was measured in winter, spring, and autumn over two years. Variation in cold tolerance was observed among populations in all three seasons, providing insight into cold adaptation within this species. Relatively high levels of cold tolerance in populations from colder environments may prove to be especially important if management strategies such as species rescue assisted migration are employed to shift populations north.

## 2 Methods

### 2.1 Data Collection

**FIGURE 1:**
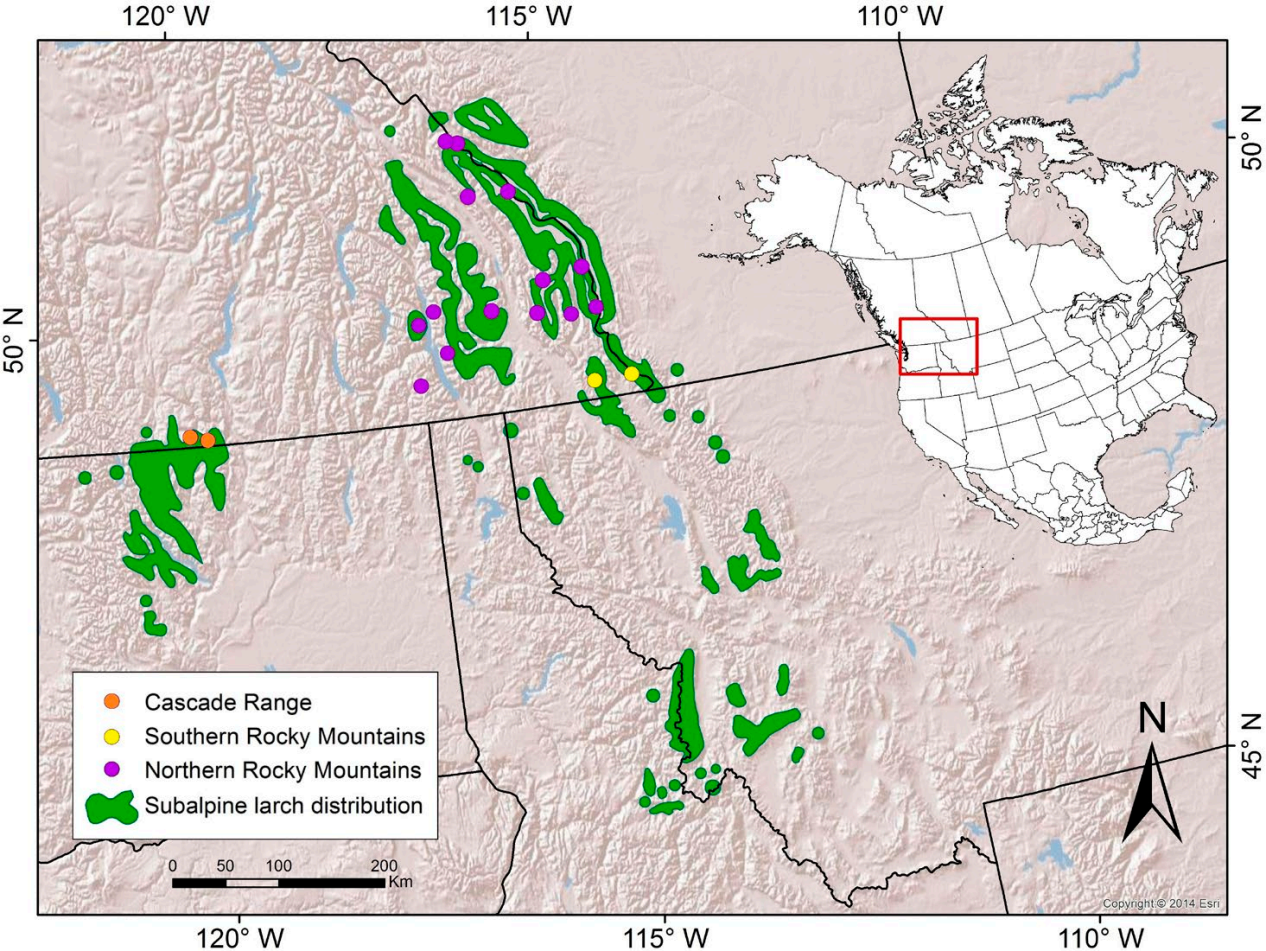
Eighteen populations of subalpine larch from the northern portion of the species range belong to three neutral genetic clusters (Vance et al. 2024): The Cascade Range (orange), the southern Rocky Mountains (yellow), and the northern Rocky Mountains (purple).

Cold tolerance in subalpine larch was assessed for 100 trees grafted *ex situ* at the Kalamalka Forestry Centre in Vernon, BC (Table 1). These trees originated from 18 populations across the Canadian portion of the species' range with representation from three neutral genetic clusters identified in a previous study (Figure 1; Vance et al. 2024). Scion collection and grafting were carried out during the winter of 1995. Scion was collected by helicopter from the upper third of the crown of adult dominant trees separated by at least 50 m (Khasa et al. 2006).

**TABLE 1:** Locations of 18 populations of subalpine larch from the Canadian portion of the species' range and the number of samples grafted *ex situ* at the Kalamalka Forestry Centre that were measured for cold tolerance (N).

Population	Location	Latitude	Longitude	Elevation (m)	N
AL01	Baldy Mountain, BC	49°19′	117°04′	1981	6
AL02	Burdett Peak—Gray Pass, BC	49°34′	116°40′	2134	6
AL03	Mount Kaslo, BC	49°55′683″	116°46′86″	2149	6
AL04	Fletcher Creek, BC	49°49′803″	116°59′694″	2012	5
AL05	Inverted Ridge, BC	49°09′433″	114°49′595″	2164	6
AL06	Sunkist Mountain, BC	49°09′722″	114°20′572″	2210	5
AL07	Racehorse Pass, BC	49°46′346″	114°39′644″	2210	5
AL08	Mount Kuleski, BC	49°44′766″	114°59′759″	2179	6
AL09	Mount Dingley, BC	49°47′834″	115°25′872″	2195	5
AL10	Mount Gass, BC	50°07′849″	114°45′770″	2256	6
AL11	Mount Mike, BC	50°03′962″	115°17′833″	2377	6
AL12	Luxor Pass—Mount Crook, BC	50°51′454″	116°07′336″	2164	5
AL13	Mount Assiniboine, BC	50°51′260″	115°34′936″	2210	6
AL14	Mount Bradford, BC	49°52′224″	116°01′463″	2454	6
AL15	Twin Buttes, BC	49°05′36″	120°06′45″	2270	6
AL16	Lake O'Hara, BC	51°21′	116°19′	2200	6
AL17	Moraine Lake, AB	51°19′	116°10′	2200	4
AL20	Harry Lake, BC	49°03′	119°53′	2200	5

Cold tolerance was measured across three seasons—winter, spring, and autumn—over two consecutive years. Temperature data from a weather station in an adjacent research trial (Hobo Micro Station, Onset Computer Corporation, Bourne, USA) were used to compare sampling conditions across years (Figures S1–S3). Conditions were found to be similar in winter but varied in the spring and autumn. Winter sampling was carried out during the last week of December in both years. Subzero temperatures were first recorded in late October and minimum daily temperatures dropped below zero for 40 days prior to sampling in both 2015 and 2016. Spring sampling was carried out on March 30th, 2015, and March 14th, 2016. In 2015, three trees had already begun to flush at the time of sampling. However, all samples were retained for analysis because spring cold tolerance is ultimately a measure of the relative timing of dehardening. Growing degree days (GDD) at the Kalamalka Forestry Centre were calculated using mean daily temperature and a threshold of 1.46°C [Ʃ(T_mean> 1.46_–1.46)] as per Worrall (1993). At the time of sampling in 2015 and 2016, 215 and 111 GDD were recorded, respectively. Finally, autumn samples were collected in mid‐October in both years. Minimum daily temperature was used to calculate hardening degree days (HDD) with a threshold of 14.5°C (Carles et al. 2012). Between August 31st and the time of sampling, 322 and 336 HDD were accumulated in the two sampling years.

Freezing tests were carried out using new growth collected from the branch tips of each tree. Stem tissue was cut into 16 pieces, each 0.5 cm in length, and distributed equally into four vials. Five drops of distilled water were added to each vial to prevent desiccation. Vials were sealed and divided between four trays, each representing a different freezing treatment, with vial position randomized within tray. The unfrozen control tray was held at 4°C while the other three trays were placed in a programmable freezer set to 0°C (Caltec Scientific Ltd., Richmond, Canada). Cold tolerance was assessed after exposure to three different sub‐zero temperatures: −20°C, −30°C, and −40°C. The freezer was cooled at a rate of five degrees per hour and held constant at each experimental freezing temperature for one hour before one tray was removed. After freezing, samples were moved to 4°C to thaw overnight. Note that in the spring of 2015, the −40°C treatment was replaced by a −10°C treatment due to expectations that flushing trees would be more prone to injury.

Electrolyte leakage was measured to assess freezing damage. After thawing, 10 mL of distilled water was added to each vial. Trays were placed on a shaker table set to 90 rpm for 24 h. A conductivity probe (4020 conductivity meter, Jenway Ltd., Dunmow, UK) was used to measure electrolyte leakage. Trays were then transferred to an oven and heated to 100°C to ensure cellular rupture. Trays were removed from the oven and kept on the shaker overnight at room temperature. Conductivity was measured again the following day.

### 2.2 Data Analysis

For each sample, relative conductivity (RC) was calculated as:

(1) $$\begin{array}{c} \mathrm{RC}=\\ \left(\text{Electrical conductivity before boiling}/\text{Electrical conductivity after boiling}\right) \end{array}$$

The index of cold injury (CI) was then calculated according to Flint et al. (1967) as follows:

(2) $$\mathrm{CI}=\left[\left({\mathrm{RC}}_{\text{freezing test}}–{\mathrm{RC}}_{\text{control}}\right)/\left(1–{\mathrm{RC}}_{\text{control}}\right)\right]$$

Note that higher CI indicated less cold tolerance. Unfortunately, CI data were not successfully collected for all samples. Branches without living cambium were collected on a few occasions and could not be assessed. Because subalpine larch is deciduous, it is not always obvious when a branch has died. Cracked lids and spillage led to the exclusion of additional samples (Table 2). Negative CI values, which arise when more electrolyte leakage occurs in controls than in frozen tissue, were converted to zero values, indicating that no damage from freezing was incurred (Table S2). Note that no negative CI values were observed in the −40°C treatment.

**TABLE 2:** Number of subalpine larch samples assessed for cold tolerance across 2 years, three seasons and four treatments out of a maximum of 100 per cell.

Year	Season	Control	Freezing temperature (°C)			
		4°C	−10	−20	−30	−40
2015	Winter	100	—	100	100	100
	Spring	100	99	99	99	—
	Autumn	99	—	92	96	88
2016	Winter	100	—	99	99	99
	Spring	98	—	97	97	98
	Autumn	99	—	85	99	99

CI data were analyzed separately by season and freezing temperature. All analyses were carried out using R version 4.4.1 (R Core Team 2024). Linear mixed effects models were fit using a Restricted Maximum Likelihood (REML) approach in ASReml‐R version 4.0 (The VSNi Team 2023). The model fit was as follows:

(3) $$Y_{\mathrm{ijk}}=\mu +P_{j}+D_{k}+{\left(P\times D\right)}_{\mathrm{jk}}+T{\left(P\right)}_{\mathrm{ij}}+\epsilon_{\mathrm{ijk}}$$

where Y_*ijk*_ is the phenotypic observation corresponding to *i*th tree (T) from the *j*th population (P) measured in the *k*th year (D); 𝜇 is the global phenotypic mean (fixed intercept) and 𝜖_*ijk*_ is the random residual error term. Population, year and their interaction (P × D) were fit as fixed effects while tree‐within‐population [T(P)] was fit as a random effect. Note that the spring analyses of the −10°C and the −40°C freezing tests did not include sampling year or individual tree as predictors because data were only collected for one year. Normality and homoscedasticity were assessed visually by plotting residuals and residuals versus fitted values, respectively. Autumn data were square‐root transformed to better meet the assumptions of the models. Because population was not a significant predictor of CI at −10°C or −20°C in any season, subsequent analysis focused on data from −30°C to −40°C trials. Wald chi‐squared tests were used to determine the significance of fixed effects. Model simplification led to the removal of year from the spring −30°C analysis and the population‐by‐year interaction from the spring −30°C and winter −40°C analyses. Differentiation among populations was further characterized by fitting a modified version of Equation (3) with all independent variables set as random and calculating the ratio of among‐population variance to total phenotypic variance (Alberto et al. 2013).

To assess phenotypic clines, population best linear unbiased estimates (BLUEs) were extracted from Equation (3) fitted models. Climate normals (1961–1990) were obtained from ClimateNA version 7.2 (Wang et al. 2016) based on the latitude, longitude, and elevation of each population. Geographic variables were combined with nineteen annual climate variables and one seasonal climate variable, autumn minimum temperature, for analysis (Table S1). Simple linear regressions were fit to test associations between population CI BLUEs and each independent variable. For this analysis, regression coefficients were considered significant at *p* < 0.002 after Bonferroni correction to account for multiple testing. Backward stepwise multiple linear regression was carried out using a model fit with the climate variable that had the highest coefficient of determination plus the three geographic variables (latitude, longitude, and elevation) as independent variables to explore the relative importance of climate versus geography in shaping CI clines in subalpine larch. Finally, Pearson correlations among population BLUEs were calculated across seasons and freezing temperatures to assess stability.

## 3 Results

**FIGURE 2:**
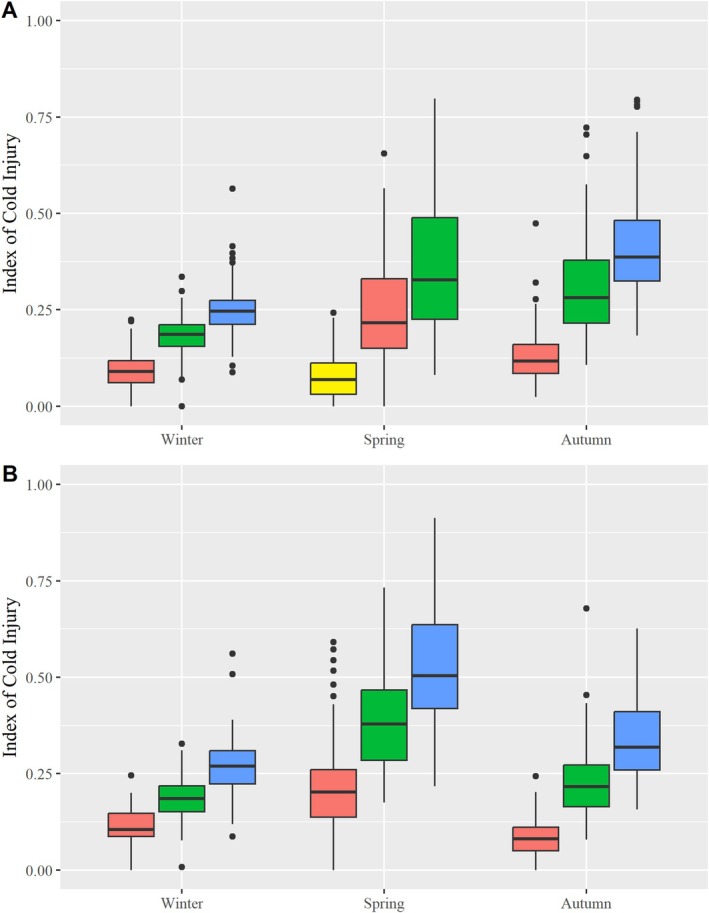
Cold injury for 100 subalpine larch trees grafted *ex situ* at the Kalamalka Forestry Centre, frozen at four different subzero temperatures (yellow = −10°C; red = −20°C; green = −30°C; blue = −40°C) in three different seasons in 2015 (A) and 2016 (B).

Cold injury varied across seasons and freezing temperatures (Figure 2). Both mean CI and the variation among individuals were highest in the spring and lowest in the winter. Within seasons, higher CI values were observed at lower subzero temperatures, as expected.

Population differentiation emerged at lower subzero temperatures (Table 3). Population was not a significant predictor of CI in the −10°C and −20°C freezing tests (not shown), nor the winter −30°C freezing test, likely due to the low levels of overall damage incurred in those experiments. However, population was a significant predictor of CI at −30°C in spring and autumn, and a significant predictor of CI at −40°C in all seasons. The proportion of phenotypic variance among populations (V_POP_) was generally low (0.08–0.15) but within the range of previously published estimates (Alberto et al. 2013). In winter, year was also a significant predictor of CI in the −40°C freezing test, with approximately 2% more damage in 2016. In autumn, year and the population‐by‐year interaction were both significant, with 6%–7% more damage in 2015. This could reflect the lower number of hardening degree days accumulated in the first year of sampling.

**TABLE 3:** Asreml linear mixed effects model outputs from analyses of cold injury in subalpine larch stem tissue measured at two different freezing temperatures (−30°C and −40°C) over three seasons (winter, spring, autumn) and two years.

	Winter		Spring		Autumn	
	−30°C	−40°C	−30°C	−40°C	−30°C	−40°C
Log‐Likelihood	377.488	374.723	235.673	102.291	282.346	269.747
AIC	−750.977	−745.446	−467.345	−202.582	−560.693	−535.494
Population *p* value	0.498	**0.001**	**0.011**	**0.012**	**< 0.001**	**< 0.001**
Year *p* value	0.833	**0.033**	0.151	NA	**< 0.001**	**< 0.001**
Pop:Year *p* value	0.106	0.932	0.403	NA	**0.002**	**0.004**
V_POP_	—	0.12 ± 0.07	0.09 ± 0.06	0.15 ± 0.10	0.10 ± 0.09	0.0843 ± 0.0842

*Note:* Wald chi‐squared tests were used to calculate the *p*‐values of fixed effects. Significance is indicated in bold. Fit metrics reflect final models after simplification. The proportion of variance among populations relative to the total phenotypic variance (V_POP_) was calculated after specifying significant fixed effects as random.

**FIGURE 3:**
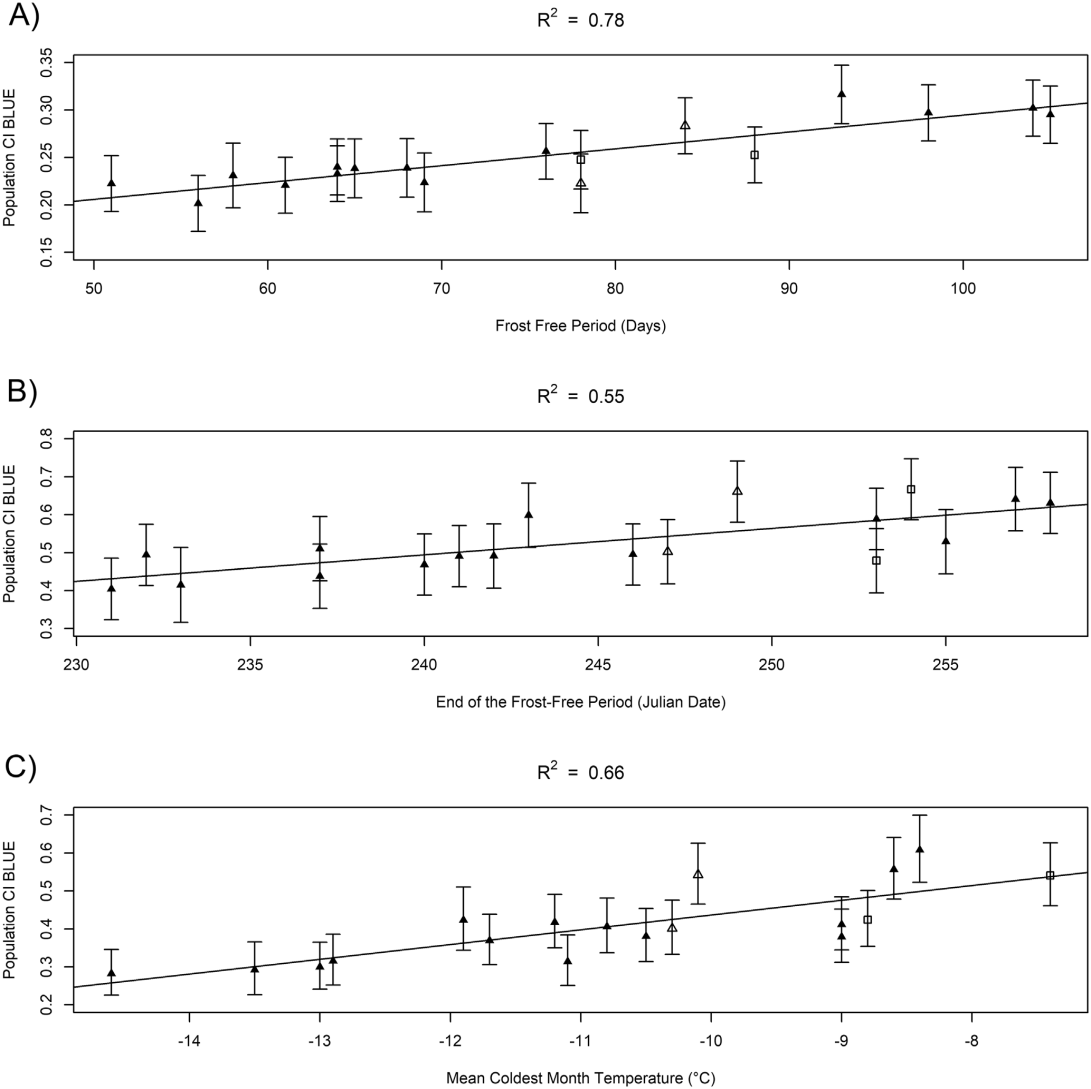
Best linear unbiased estimates (BLUEs with standard errors) for cold injury (CI) at −40°C exhibit phenotypic clines along climatic gradients in winter (A), spring (B), and autumn (C) for populations of subalpine larch from the northern Rocky Mountains (black triangles), southern Rocky Mountains (open triangles), and Cascade Range (open squares). Note that BLUEs and standard errors for transformed autumn CI data were rescaled prior to plotting.

Strong phenotypic clines were observed in all three seasons (Figure 3). Significant phenotype‐by‐environment interactions were not detected in winter or spring at −30°C but strong clines emerged in autumn (Table S3) and at −40°C in all seasons (Table 4). In winter, nine temperature‐associated climate variables were significant predictors of population CI at −40°C and the length of the frost‐free period (FFP) was the strongest (*R* ^2^ = 0.78). In spring, five temperature‐associated climate variables were significant predictors of population CI at −40°C and the Julian date on which the FFP ends (eFFP) was the strongest (*R* ^2^ = 0.55). In autumn, latitude plus eight temperature‐associated variables were significant predictors of population CI at −30°C and seven temperature‐associated variables were significant predictors of population CI at −40°C; mean coldest month temperature (MCMT) was the strongest predictor of autumn population CI at both −30°C and −40°C (*R* ^2^ = 0.68 and 0.66, respectively). Backward stepwise multiple linear regression found that geographic variables did not improve model fits for the climate variables with the highest coefficients of determination and were therefore discarded. Note that FFP, eFFP and MCMT were significant predictors of population CI at −40°C in all three seasons and were all highly correlated across the populations included in this study (> 0.86). Across seasons, populations with lower CI and therefore higher cold tolerance were from colder sites with a shorter FFP.

**TABLE 4:** Three geographic variables and twenty climate variables (defined in Table S1) were used to identify phenotypic clines in cold injury across 18 populations of subalpine larch.

Climate variable	Climatic variation		Winter −40°C			Spring −40°C			Autumn −40°C		
	Low	High	*p*	*R* ^2^	Slope	*p*	*R* ^2^	Slope	*p*	*R* ^2^	Slope
Latitude (dd)	49.050	51.350	0.051	0.22	−0.0214	0.010	0.35	−0.0674	0.012	0.33	−0.0590
Longitude (dd)	−120.107	−114.343	0.298	0.07	−0.0054	0.196	0.1	−0.0165	0.112	0.15	−0.0180
Elevation (m)	1981	2454	0.004	0.41	−0.0002	0.139	0.13	−0.0003	0.324	0.06	−0.0002
MAT (°C)	−3.4	1.1	**0.001**	**0.51**	**0.0197**	0.013	0.33	0.0393	**< 0.002**	**0.47**	**0.0419**
MWMT (°C)	8.1	12.3	0.016	0.31	0.0156	0.234	0.09	0.0205	0.120	0.14	0.0236
MCMT (°C)	−14.6	−7.4	**0.001**	**0.51**	**0.0121**	**0.001**	**0.50**	**0.0297**	**< 0.001**	**0.66**	**0.0304**
TD (°C)	17.1	24.1	0.076	0.18	−0.0083	0.008	0.36	−0.0290	0.003	0.44	−0.0285
Tmin_at (°C)	−7.1	−1.7	**< 0.001**	**0.61**	**0.0169**	**< 0.002**	**0.47**	**0.0371**	**< 0.001**	**0.56**	**0.0362**
MAP (mm)	773	1853	0.254	0.08	0.0000	0.449	0.04	0.0001	0.965	0.00	0.0000
MSP (mm)	242	681	0.092	0.17	−0.0001	0.080	0.18	−0.0003	0.024	0.28	−0.0003
AHM (°C/μm)	4.5	13.3	0.718	0.01	0.0013	0.474	0.03	0.0066	0.171	0.11	0.0111
SHM (°C/μm)	13	40.2	0.049	0.22	0.0021	0.069	0.19	0.0049	0.017	0.31	0.0055
DD_0 (days)	1165	2140	**0.001**	**0.50**	**−0.0001**	0.004	0.41	−0.0002	**< 0.001**	**0.57**	**−0.0002**
DD_5 (days)	300	712	0.008	0.36	0.0002	0.158	0.12	0.0002	0.079	0.18	0.0003
NFFD (days)	91	150	**< 0.001**	**0.71**	**0.0017**	0.004	0.41	0.0032	0.003	0.44	0.0029
FFP (days)	51	105	**< 0.001**	**0.78**	**0.0018**	**< 0.001**	**0.552**	**0.0037**	**0.001**	**0.49**	**0.0031**
bFFP (date)	152	181	**< 0.001**	**0.68**	**−0.0032**	0.003	0.44	−0.0063	0.020	0.29	−0.0046
eFFP (date)	231	258	**< 0.001**	**0.69**	**0.0031**	**< 0.001**	**0.554**	**0.0070**	**< 0.001**	**0.59**	**0.0065**
PAS (mm)	503	1238	0.188	0.11	0.0001	0.268	0.08	0.0001	0.880	0.00	0.0000
EMT (°C)	−50	−37.4	**0.001**	**0.53**	**0.0066**	**< 0.002**	**0.48**	**0.0156**	**< 0.001**	**0.59**	**0.0156**
EXT (°C)	25.5	29.5	0.112	0.15	0.0105	0.580	0.02	0.0094	0.303	0.07	0.0155
Eref (mm)	287	421	0.782	0.00	0.0001	0.695	0.01	−0.0002	0.696	0.01	0.0002
CMD (mm)	0	80	0.657	0.01	0.0002	0.362	0.05	0.0008	0.157	0.12	0.0011

*Note:* Significance is indicated in bold.

Population CI BLUEs were remarkably stable across seasons and freezing temperatures. Strong, positive correlations were observed across all three seasons (0.66–0.69) in the −40°C freezing tests. Within seasons, very strong correlations were observed between the −30°C and −40°C freezing tests in spring (0.76) and autumn (0.94) but not in winter (0.17).

## 4 Discussion

Subalpine larch exhibits local adaptation for cold tolerance within the northern portion of its range. Although treeline is often described as a relatively consistent environment (Richardson and Friedland 2009) and the 18 populations of subalpine larch included in this study occupy a restricted latitudinal range (49.05°N–51.35°N), populations from colder environments with shorter frost‐free periods were found to have significantly higher cold tolerance in all three seasons (winter, spring, and autumn), suggesting that environmental differentiation has shaped patterns of phenotypic variation. Because subalpine larch populations are so difficult to access—being both remote and snowed in for a significant portion of the year—this is the first evidence of local adaptation for any trait in this species.

Cold tolerance appears to have evolved in response to temperature‐associated climatic variation. Populations with higher winter cold tolerance came from colder sites with significantly lower mean annual temperatures (MAT), mean coldest month temperatures (MCMT), extreme minimum temperatures (EMT) and minimum autumn temperatures (Tmin_at), as well as more degree days below zero (DD_0). These sites also had shorter frost‐free periods that started later (bFFP) and ended earlier (eFFP) with fewer frost‐free days (NFFD). The length of the frost‐free period (FFP) was the strongest predictor of population winter cold tolerance. FFP is strongly correlated with both latitude and elevation across the populations included in this study (−0.60 and −0.76), but these two geographic variables are only weakly correlated with each other (0.10), and stepwise backward multiple regression found that the inclusion of geographic variables did not improve the fit of the FFP model. This suggests that populations of subalpine larch are adapting to local conditions on their respective mountaintops. Local adaptation evolves when the strength of selection is greater than the rate of migration (Haldane 1930). Across populations, FFP varied by 43 days, MAT varied by 4.5°C, and elevation varied by 473 m. Such heterogeneity could impose strong selection. Furthermore, pollen dispersal in the Rocky Mountains may be limited by prevailing winds from the southwest in combination with rugged topography. In a closely related species, western larch ( *Larix occidentalis* ), genetic differentiation between populations from the same geographic areas was observed at elevational intervals of approximately 500 m (Rehfeldt 1995).

Genetic variation for midwinter cold tolerance is not often observed (Howe et al. 2003). For example, a range‐wide study of another high‐elevation conifer species, whitebark pine ( *Pinus albicaulis* ), found no regional differences in winter cold injury after needles were frozen at −62°C (Bower and Aitken 2006). Variation in winter cold tolerance is not expected because most conifers develop midwinter hardiness that far exceeds the minimum temperatures that they experience. For example, Sakai and Weiser (1973) found that the leaves of two of subalpine larch's most common associates, subalpine fir ( *Abies lasiocarpa* ) and Engelmann spruce ( *Picea engelmannii* ), were not visibly damaged after freezing at −80°C and −70°C, respectively, although they both suffered bud damage when exposed to temperatures lower than −40°C. Subalpine larch is hardier than either of these species. The average extreme minimum temperature for the populations included in this study is −42°C but drops as low as −50°C at Lake O'Hara (2200 m). At their study site in Marmot Valley, Alberta, Richards and Bliss (1986) found that subalpine larch suffered significantly less winter damage than sympatric conifers, sustaining two thirds less damage than subalpine fir, the second hardiest species. It is therefore possible that subalpine larch grafts growing at the Kalamalka Forestry Centre were not fully acclimated. Cold acclimation is triggered by long nights and then by low temperatures but it has been suggested that some species may undergo a third stage of acclimation after exposure to very low temperatures (< −30°C; Weiser 1970). Daily minimum temperatures at Kalamalka stayed above −20°C prior to sampling in both years. Alternately, muted variation in winter cold injury (0.20–0.32 across populations) at levels well below that at which tissue damage or death is expected (0.50) may reflect more subtle differences in cellular physiology (Strimbeck et al. 2015). Differences in lipid composition, oligosaccharide accumulation, and/or dehydrin accumulation could generate variable levels of moderate, sub‐lethal electrolyte leakage across the plasma membrane. Further studies on the biochemical basis of cold acclimation in subalpine larch are needed to elucidate this issue.

Higher levels of freezing damage were observed in the spring and autumn. Late spring frosts and early autumn frosts can cause significant damage during phases of active growth as conifers undergo deacclimation and acclimation, respectively. Variation in cold tolerance therefore reflects variation in the timing of these processes. In this study, the Julian date for the end of the frost‐free period (eFFP) and the mean coldest month temperature (MCMT) were the strongest predictors of spring and autumn cold tolerance, respectively, although these climate variables were significant in both seasons. Populations from colder environments with shorter frost‐free periods had higher cold tolerance. Both eFFP and MCMT are strongly correlated with latitude (−0.80 and −0.83) and moderately correlated with longitude (−0.48 and −0.54), but it is unlikely that the observed patterns of phenotypic variation arose via neutral processes related to dispersal. A recent analysis of 751 neutral SNPs identified three genetically differentiated regions in the Canadian portion of the species range: the Cascade Range, the southern Rocky Mountains, and the northern Rocky Mountains (Vance et al. 2024). Populations from all three regions were included in this study but cold tolerance phenotypes did not cluster by region. Instead, patterns of phenotypic variation appear to follow correlated climatic and geographic gradients.

Adaptive clines for cold tolerance traits have been observed in many conifer species (Aitken and Hannerz 2001; Howe et al. 2003; Alberto et al. 2013). It is well established that trees from colder environments with shorter growing seasons develop cold tolerance earlier to avoid frost damage in autumn. For example, latitude and MCMT were both significant predictors of regional cold tolerance in whitebark pine when needles were frozen at −40°C in autumn (Bower and Aitken 2006). Trees from higher latitudes with lower MCMT had higher autumn cold tolerance, supporting the results of this study. Less commonly observed are positive correlations in cold tolerance across seasons. For example, negative genetic correlations were documented between spring and autumn cold tolerance in Douglas‐fir (O'Neill et al. 2000, 2001). This is generally attributed to the fact that trees from colder, more continental environments have lower chilling requirements, lower heat sum requirements, or a combination thereof, which allows them to break dormancy and initiate budburst earlier in common garden experiments (Aitken and Hannerz 2001). Trees from warmer environments have higher chilling and/or heat‐sum requirements to avoid prematurely initiating growth during warm spells. However, delays in budburst have previously been documented for populations of western larch from higher elevations (Rehfeldt 1982) and more continental environments (Roskilly and Aitken 2024), supporting the results of this study. These patterns could indicate a failure to meet chilling requirements on relatively mild test sites. Subalpine larch and western larch are known to have low heat‐sum requirements when chilling requirements are met (Worrall 1993; Harrington and Gould 2015) but heat‐sum requirements increase exponentially in western larch and eastern larch ( *Larix laricina* ) when chilling requirements are not met (Harrington and Gould 2015; Man et al. 2021). Anecdotally, grafted subalpine larch trees at the Kalamalka Forestry Centre always flush later than grafted western larch trees in a nearby clone bank, suggesting that subalpine larch has a higher chilling requirement and/or a higher forcing requirement than its low‐elevation relative within this environmental context. It would make sense for subalpine larch to have a higher chilling and/or forcing requirement than western larch based on its habitat. At treeline, subalpine larch experiences strong selection against responding prematurely to warm daytime temperatures in early spring. This could equally apply to populations of subalpine larch from colder environments. The populations included in this study experience subzero temperatures for eight to ten months of the year (September–May) with a frost‐free period that can start as late as July 1st and end as early as August 19th. Maintaining cold tolerance during high‐risk periods is necessary to avoid injury. Local adaptation for cold tolerance traits is therefore the key to subalpine larch's success at treeline in the Cascade Range and Rocky Mountains.

Understanding patterns of adaptive variation is crucial for managing forest genetic resources. A previous study found lower genetic diversity within and higher genetic differentiation among populations of subalpine larch compared to western larch, which has a more contiguous distribution at lower elevations (Khasa et al. 2006). This was attributed to genetic bottlenecks during post‐Pleistocene range expansion as well as ongoing genetic drift within populations subdivided by the fragmentation of suitable high‐elevation habitat. However, the results of the current study indicate that genetic differentiation in subalpine larch has also been driven by natural selection acting on key traits such as cold tolerance. Both neutral and selective pressures can shape patterns of genetic variation during range expansions (White et al. 2013). When subalpine larch migrated north following the retreat of the Cordilleran ice sheet, northern environments could have imposed strong selection for cold adaptation traits. Even today, environmental differentiation persists across the Canadian portion of the species range. Focusing on neutral genetic structure to guide management strategies would therefore preserve some of the phenotypic variation observed in this study, but populations with different levels of seasonal cold tolerance should also be prioritized for conservation. Higher levels of cold tolerance may prove to be especially valuable if management strategies such as species rescue assisted migration (AM) are employed.

Species rescue AM involves the intentional movement of species that are threatened by climate change but have low migration potential (Pedlar et al. 2012). Subalpine larch meets these qualifications and is also a good candidate for species rescue AM because it is slow‐growing and unlikely to become invasive if planted outside of its current range. Species distribution models could be used to identify appropriate sites for planting but, for subalpine larch, it would most likely mean moving trees large distances northward (pers. comm. Dr. Tongli Wang). Seed would need to be collected from target populations, which could be identified based on both the neutral genetic structure of the species as well as local adaptation for cold tolerance. Cold‐origin populations may be particularly important if their relatively high cold tolerance gives them an advantage at high latitudes. Common‐garden provenance or progeny tests could be established both as an inter‐situ conservation resource and to study adaptive traits such as cold tolerance, growth, and disease resistance (Yanchuk 2001). For subalpine larch, active management strategies such as assisted migration could thus provide multiple benefits to buffer against predicted climate change and possible maladaptation.

## Conflicts of Interest

The authors declare no conflicts of interest.

## Supporting information


**Figure S1:** Maximum (plum) and minimum (turquoise) daily temperature at the Kalamalka Forestry Centre in Vernon, BC, from October 1st until tissue was sampled from subalpine larch trees (dashed lines) on December 30th, 2014, (A) and December 29, 2015 (B).
**Figure S2:** Maximum (plum) and minimum (turquoise) daily temperature at the Kalamalka Forestry Centre in Vernon, BC, from January 1st until tissue was sampled from subalpine larch trees (dashed lines) on March 31st, 2015, (A) and March 15th, 2016 (B).
**Figure S3:** Maximum (plum) and minimum (turquoise) daily temperature at the Kalamalka Forestry Centre in Vernon, BC, from August 1st until tissue was sampled from subalpine larch trees (dashed lines) on October 19th, 2015, (A) and October 17th, 2016 (B).

**Table S1:** Nineteen annual climate variables and one seasonal climate variable used to study local adaptation in subalpine larch.

**Table S2:** Number of individuals with higher electrolyte leakage in controls than in frozen samples for cold injury assessment of 100 subalpine larch trees frozen at different sub‐zero temperatures.

**Table S3:** Three geographic variables and twenty climate variables (defined in Table S1) were used to identify phenotypic clines in cold injury across 18 populations of subalpine larch after freezing at −30 degrees C. Significance is indicated in bold.

## Data Availability

The data that support the findings of this study are available from the corresponding author upon reasonable request.

## References

[eva70201-bib-0001] Aitken, S. N. , and M. Hannerz . 2001. “Genecology and Gene Resource Management Strategies for Conifer Cold Hardiness.” In Conifer Cold Hardiness, edited by F. J. Bigras and S. J. Colombo , 23–53. Kluwer Academic Publishers.

[eva70201-bib-0002] Aitken, S. N. , S. Yeaman , J. A. Holliday , T. Wang , and S. Curtis‐McLane . 2008. “Adaptation, Migration or Extirpation: Climate Change Outcomes for Tree Populations.” Evolutionary Applications 1: 95–111. 10.1111/j.1752-4571.2007.00013.x. 25567494 PMC3352395

[eva70201-bib-0003] Alberto, F. J. , S. N. Aitken , R. Alía , et al. 2013. “Potential for Evolutionary Response to Climate Change—Evidence From Tree Populations.” Global Change Biology 19: 1645–1661. 10.1111/gcb.12181. 23505261 PMC3664019

[eva70201-bib-0004] Arno, S. F. , and J. R. Habeck . 1972. “Ecology of Alpine Larch ( *Larix lyallii* Parl.) in the Pacific Northwest.” Ecological Monographs 42, no. 4: 417–450. 10.2307/1942166.

[eva70201-bib-0005] Berg, E. E. , and F. S. I. I. I. Chapin . 1994. “Needle Loss as a Mechanism of Winter Drought Avoidance in Boreal Conifers.” Canadian Journal of Forest Research 24: 1144–1148. 10.1139/x94-151.

[eva70201-bib-0006] Bower, A. D. , and S. N. Aitken . 2006. “Geographic and Seasonal Variation in Cold Hardiness of Whitebark Pine.” Canadian Journal of Forest Research 36: 1842–1850. 10.1139/X06-067.

[eva70201-bib-0007] Carles, S. , M. S. Lamhamedi , D. C. Stowe , L. Veilleux , and H. A. Margolis . 2012. “An Operational Method for Estimating Cold Tolerance Thresholds of White Spruce Seedlings in Forest Nurseries.” Forestry Chronicle 88, no. 4: 448–456. 10.5558/tfc2012-08.

[eva70201-bib-0008] Flint, J. L. , B. R. Boyce , and D. J. Beattie . 1967. “Index of Injury—A Useful Expression of Freezing Injury to Plant Tissues as Determined by the Electrolytic Method.” Canadian Journal of Plant Science 47: 229–230. 10.4141/cjps67-043.

[eva70201-bib-0009] Gower, S. T. , and J. H. Richards . 1990. “Larches: Deciduous Conifers in an Evergreen World.” Bioscience 40, no. 1: 818–826. 10.2307/1311484.

[eva70201-bib-0010] Haldane, J. B. S. 1930. “A Mathematical Theory of Natural and Artificial Selection (Part VI, Isolation).” Mathematical Proceedings of the Cambridge Philosophical Society 26: 220–230. 10.1017/S0305004100015450.

[eva70201-bib-0011] Hamrick, J. L. , M. J. W. Godt , and S. L. Sherman‐Broyles . 1992. “Factors Influencing Levels of Genetic Diversity in Woody Plant Species.” New Forests 6: 95–124. 10.1007/BF00120641.

[eva70201-bib-0012] Harrington, C. A. , and P. J. Gould . 2015. “Tradeoffs Between Chilling and Forcing Satisfying Dormancy Requirements for Pacific Northwest Tree Species.” Frontiers in Plant Science 6, no. 120: 1–12. 10.3389/fpls.2015.00120. 25784922 PMC4347443

[eva70201-bib-0013] Howe, G. T. , S. N. Aitken , D. B. Neale , K. D. Jermstad , N. C. Wheeler , and T. H. H. Chen . 2003. “From Genotype to Phenotype: Unravelling the Complexities of Cold Adaptation in Forest Trees.” Canadian Journal of Botany 81: 1247–1266. 10.1139/b03-141.

[eva70201-bib-0014] Intergovernmental Panel on Climate Change . 2013. Climate Change 2013: The Physical Science Basis. Contribution of Working Group I to the Fifth Assessment Report of the Intergovernmental Panel on Climate Change, edited by T. F. Stocker , D. Qin , G.‐K. Plattner , et al. Cambridge University Press.

[eva70201-bib-0017] Khasa, D. P. , J. P. Jaramillo‐Correa , B. Jaquish , and J. Bousquet . 2006. “Contrasting Microsatellite Variation Between Subalpine and Western Larch, Two Closely Related Species With Different Distribution Patterns.” Molecular Ecology 15: 3907–3918. 10.1111/j.1365-294X.2006.03066.x. 17054492

[eva70201-bib-0018] Lynch, M. , and R. Lande . 1993. “Evolution and Extinction in Response to Environmental Change.” In Biotic Interactions and Global Change, edited by P. Kareiva , J. Kingsolver , and R. Huey , 234–250. Sinauer Assoc., Inc.

[eva70201-bib-0019] MacLachlan, I. R. , T. Wang , A. Hamann , P. Smets , and S. N. Aitken . 2017. “Selective Breeding of Lodgepole Pine Increases Growth and Maintains Climatic Adaptation.” Forest Ecology and Management 391: 404–416. 10.1016/j.foreco.2017.02.008.

[eva70201-bib-0020] Man, R. , P. Lu , and Q.‐L. Ding . 2021. “Effects of Insufficient Chilling on Budburst and Growth of Six Temperate Forest Tree Species in Ontario.” New Forests 52: 303–315. 10.1007/s11056-020-09795-1.

[eva70201-bib-0022] O'Neill, G. A. , W. T. Adams , and S. N. Aitken . 2001. “Quantitative Genetics of Spring and Fall Cold Hardiness in Seedlings From Two Oregon Populations of Coastal Douglas‐Fir.” Forest Ecology and Management 149: 305–318.

[eva70201-bib-0023] O'Neill, G. A. , S. N. Aitken , and W. T. Adams . 2000. “Genetic Selection for Cold Hardiness in Coastal Douglas‐Fir Seedlings and Saplings.” Canadian Journal of Forest Research 30: 1799–1807.

[eva70201-bib-0024] Pedlar, J. H. , D. W. McKenney , I. Aubin , et al. 2012. “Placing Forestry in the Assisted Migration Debate.” Bioscience 62: 835–842. 10.1525/bio.2012.62.9.10.

[eva70201-bib-0025] Porth, I. , and Y. A. El‐Kassaby . 2014. “Assessment of the Genetic Diversity of Forest Tree Populations Using Molecular Markers.” Diversity 6: 283–295. 10.3390/d6020283.

[eva70201-bib-0026] R Core Team . 2024. R: A Language and Environment for Statistical Computing. R Foundation for Statistical Computing. https://www.R‐project.org/.

[eva70201-bib-0027] Rehfeldt, G. E. 1982. “Differentiation of *Larix occidentalis* Populations From the Northern Rocky Mountains.” Silvae Genetica 319, no. 1: 13–19.

[eva70201-bib-0028] Rehfeldt, G. E. 1995. “Genetic Variation, Climate Models and the Ecological Genetics of *Larix occidentalis* .” Forest Ecology and Management 78: 21–37. 10.1016/0378-1127(95)03602-4.

[eva70201-bib-0029] Richards, J. H. , and L. C. Bliss . 1986. “Winter Water Relations of a Deciduous Timberline Conifer, *Larix lyallii* Parl.” Oecologia 69, no. 1: 16–24. 10.1007/BF00399032. 28311679

[eva70201-bib-0030] Richardson, A. D. , and A. J. Friedland . 2009. “A Review of the Theories to Explain Arctic and Alpine Treelines Around the World.” Journal of Sustainable Forestry 28: 218–242. 10.1080/10549810802626456.

[eva70201-bib-0031] Roberts, D. R. , and A. Hamann . 2015. “Glacial Refugia and Modern Genetic Diversity of 22 Western North American Tree Species.” Proceedings of the Royal Society B 282: 20142903. 10.1098/rspb.2014.2903. 25761711 PMC4375868

[eva70201-bib-0032] Roskilly, B. , and S. N. Aitken . 2024. “Weak Local Adaptation to Climate in Seedlings of a Deciduous Conifer Suggests Limited Benefits and Risks of Assisted Gene Flow.” Evolutionary Applications 17: e70001. 10.1111/eva.70001. 39286764 PMC11403170

[eva70201-bib-0033] Sakai, A. , and C. J. Weiser . 1973. “Freezing Resistance of Trees in North America With Reference to Tree Regions.” Ecology 54, no. 1: 118–126. 10.2307/1934380.

[eva70201-bib-0034] Strimbeck, G. R. , P. G. Schaberg , C. G. Fossdal , W. P. Shröder , and T. D. Kjellsen . 2015. “Extreme Low Temperature Tolerance in Woody Plants.” Frontiers in Plant Science 6: 884. 10.3389/fpls.2015.00884. 26539202 PMC4609829

[eva70201-bib-0035] The VSNi Team . 2023. “asreml: Fits Linear Mixed Models Using REML. R Package Version 4.2.0.332.” www.vsni.co.uk.

[eva70201-bib-0036] Vance, M. , J. Richardson , B. Hawkins , and P. von Aderkas . 2024. “The Range‐Wide Genetic Structure of a High‐Elevation Conifer Species, Subalpine Larch.” Journal of Biogeography 51: 2556–2565. 10.1111/jbi.15002.

[eva70201-bib-0037] Wang, T. , E. M. Campbell , G. A. O'Neill , and S. N. Aitken . 2012. “Projecting Future Distributions of Ecosystem Climate Niches: Uncertainties and Management Applications.” Forest Ecology and Management 279: 128–140. 10.1016/j.foreco.2012.05.034.

[eva70201-bib-0038] Wang, T. , A. Hamann , D. L. Spittlehouse , and T. Q. Murdock . 2016. “ClimateWNA—High‐Resolution Spatial Climate Data for Western North America.” Journal of Applied Meteorology and Climatology 51: 16–29. 10.1175/JAMC-D-11-043.1.

[eva70201-bib-0039] Weiser, C. J. 1970. “Cold Resistance and Injury in Woody Plants.” Science 169, no. 3952: 1269–1278. 10.1126/science.169.3952.1269. 17772511

[eva70201-bib-0040] White, T. A. , S. E. Perkins , G. Heckel , and J. B. Searle . 2013. “Adaptive Evolution During an Ongoing Range Expansion: The Invasive Bank Vole ( *Myodes glareolus* ) in Ireland.” Molecular Ecology 22: 2971–2985. 10.1111/mec.12343. 23701376

[eva70201-bib-0041] Worrall, J. 1993. “Temperature Effects on Bud‐Burst and Leaf‐Fall in Subalpine Larch.” Journal of Sustainable Forestry 1, no. 2: 1–19. 10.1300/J091v01n02_01.

[eva70201-bib-0042] Yanchuk, A. D. 2001. “A Quantitative Framework for Breeding and the Conservation of Forest Tree Genetic Resources in British Columbia.” Canadian Journal of Forest Research 31: 566–576. 10.1139/x00-13.

