# Supplementary material for: Local Adaptation for Seasonal Cold Tolerance in a High‐Elevation Conifer Species, Subalpine Larch (Larix lyallii Parl.)
Source: Evol Appl. 2026 Feb 19;19(2):e70201. doi: 10.1111/eva.70201 (PMC12920685; doi:10.1111/eva.70201)
Supplement: Supplementary file 1 — Figure S1: Maximum (plum) and minimum (turquoise) daily temperature at the Kalamalka Forestry Centre in Vernon, BC, from October 1st until tissue was sampled from subalpine larch trees (dashed lines) on December 30th, 2014, (A) and December 29, 2015 (B). Figure S2: Maximum (plum) and minimum (turquoise) daily temperature at the Kalamalka Forestry Centre in Vernon, BC, from January 1st until tissue was sampled from subalpine larch trees (dashed lines) on March 31st, 2015, (A) and March 15th, 2016 (B). Figure S3: Maximum (plum) and minimum (turquoise) daily temperature at the Kalamalka Forestry Centre in Vernon, BC, from August 1st until tissue was sampled from subalpine larch trees (dashed lines) on October 19th, 2015, (A) and October 17th, 2016 (B). [file EVA-19-e70201-s003.docx]

Supplementary Information


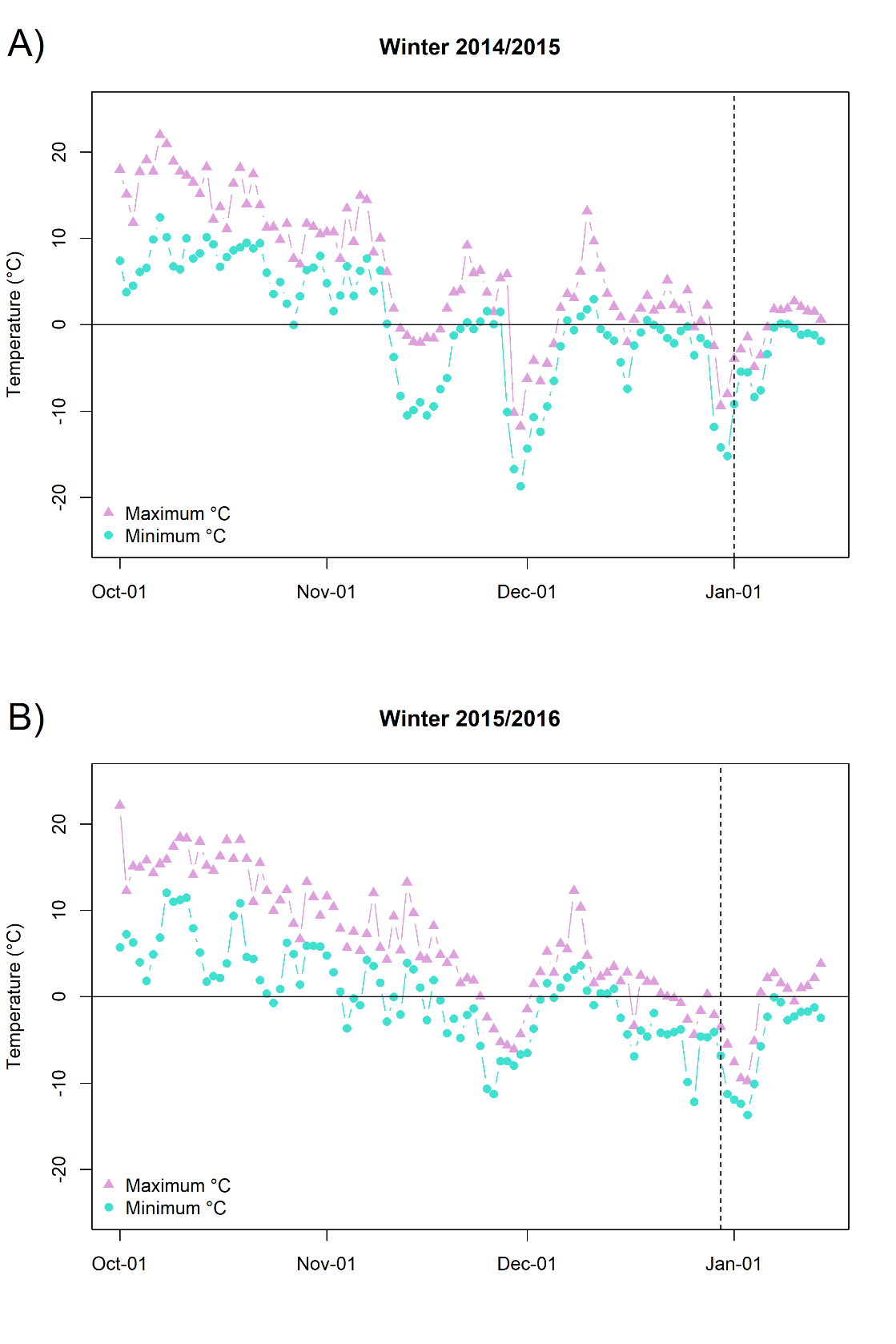


Figure 1. Maximum (plum) and minimum (turquoise) daily temperature at the Kalamalka Forestry Centre in Vernon, BC, from October 1^st^ until tissue was sampled from subalpine larch trees (dashed lines) on December 30^th^, 2014, (A), and December 29, 2015 (B).


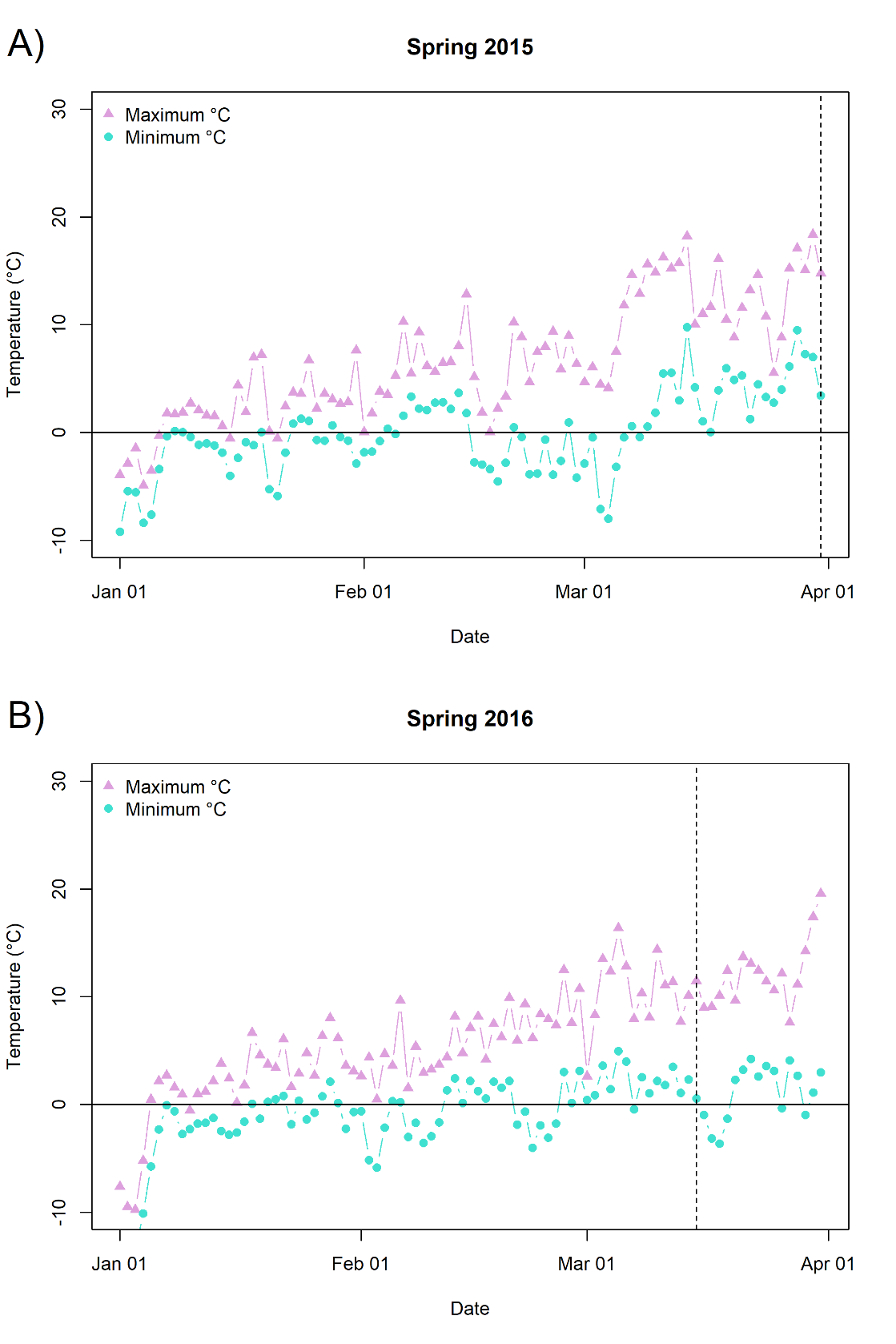


Figure 2. Maximum (plum) and minimum (turquoise) daily temperature at the Kalamalka Forestry Centre in Vernon, BC, from January 1^st^ until tissue was sampled from subalpine larch trees (dashed lines) on March 31^st^, 2015, (A), and March 15^th^, 2016 (B).


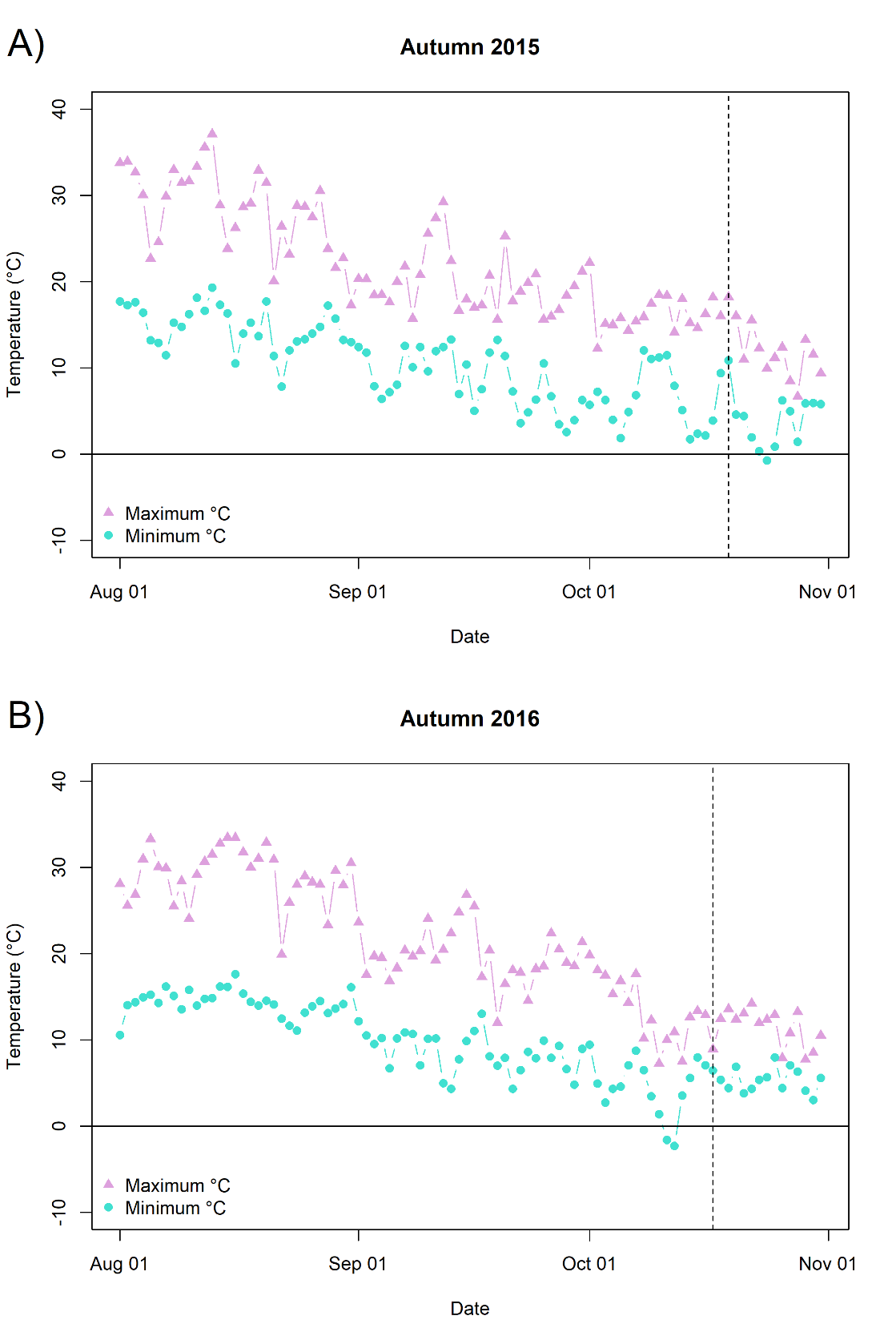


Figure 3. Maximum (plum) and minimum (turquoise) daily temperature at the Kalamalka Forestry Centre in Vernon, BC, from August 1^st^ until tissue was sampled from subalpine larch trees (dashed lines) on October 19^th^, 2015, (A), and October 17^th^, 2016 (B).
