# Supplementary material for: Local Adaptation for Seasonal Cold Tolerance in a High‐Elevation Conifer Species, Subalpine Larch (Larix lyallii Parl.)
Source: Evol Appl. 2026 Feb 19;19(2):e70201. doi: 10.1111/eva.70201 (PMC12920685; doi:10.1111/eva.70201)
Supplement: Supplementary file 2 — Table S1: Nineteen annual climate variables and one seasonal climate variable used to study local adaptation in subalpine larch. [file EVA-19-e70201-s004.docx]

Supplementary Information

Table 1. Nineteen annual climate variables and one seasonal climate variable used to study local adaptation in subalpine larch.

| Environmental Variable (unit) | Abbreviation |
| --- | --- |
| Mean annual temperature (˚C) | MAT |
| Mean warmest month temperature (˚C) | MWMT |
| Mean coldest month temperature (˚C) | MCMT |
| Continentality (MWMT minus MCMT) (˚C) | TD |
| Minimum temperature in autumn (˚C) | Tmin_at |
| Mean annual precipitation (mm) | MAP |
| May to September precipitation (mm) | MSP |
| Annual heat-moisture index (MAT + 10)/(MAP/1000) (˚C/µm) | AHM |
| Summer heat-moisture index (MWMT/(MSP/1000) (˚C/µm) | SHM |
| Degree-days below 0˚C, chilling degree days | DD_0 |
| Degree-days above 5˚C, growing-degree days | DD_5 |
| Number of frost-free days (days) | NFFD |
| Frost-free period (days) | FFP |
| The day of the year on which FFP begins (Julian date) | bFFP |
| The day of the year on which FFP ends (Julian date) | eFFP |
| Precipitation as snow between August and July (mm) | PAS |
| Extreme minimum temperature over 30 years (˚C) | EMT |
| Extreme maximum temperature over 30 years (˚C) | EXT |
| Hargreaves reference evaporation (mm) | Eref |
| Hargreaves climatic moisture deficit (mm) | CMD |
