# Supplementary material for: Local Adaptation for Seasonal Cold Tolerance in a High‐Elevation Conifer Species, Subalpine Larch (Larix lyallii Parl.)
Source: Evol Appl. 2026 Feb 19;19(2):e70201. doi: 10.1111/eva.70201 (PMC12920685; doi:10.1111/eva.70201)
Supplement: Supplementary file 3 — Table S2: Number of individuals with higher electrolyte leakage in controls than in frozen samples for cold injury assessment of 100 subalpine larch trees frozen at different sub‐zero temperatures. [file EVA-19-e70201-s002.docx]

Supplementary Information

Table 2. Number of individuals with higher electrolyte leakage in controls than in frozen samples for cold injury assessment of 100 subalpine larch trees frozen at different sub-zero temperatures

|  | Winter | Spring | Autumn |
| --- | --- | --- | --- |
| Tray -10˚C | 0 | 9 | 0 |
| Tray -20˚C | 5 | 3 | 2 |
| Tray -30˚C | 1 | 0 | 0 |
| Tray -40˚C | 0 | 0 | 0 |
