# Supplementary material for: Local Adaptation for Seasonal Cold Tolerance in a High‐Elevation Conifer Species, Subalpine Larch (Larix lyallii Parl.)
Source: Evol Appl. 2026 Feb 19;19(2):e70201. doi: 10.1111/eva.70201 (PMC12920685; doi:10.1111/eva.70201)
Supplement: Supplementary file 4 — Table S3: Three geographic variables and twenty climate variables (defined in Table S1) were used to identify phenotypic clines in cold injury across 18 populations of subalpine larch after freezing at −30 degrees C. Significance is indicated in bold. [file EVA-19-e70201-s001.docx]

Supplementary Information

Table 3. Three geographic variables and twenty climate variables (defined in Table S1) were used to identify phenotypic clines in cold injury across 18 populations of subalpine larch. Significance is indicated in bold.

| Climate Variable | Climatic Variation | | Winter -30˚C | | | Spring -30˚C | | | Autumn -30˚C | | |
| --- | --- | --- | --- | --- | --- | --- | --- | --- | --- | --- | --- |
|  | Low | High | Pval | R^2^ | Slope | Pval | R^2^ | Slope | Pval | R^2^ | Slope |
| Latitude (dd) | 49.050 | 51.350 | 0.474 | 0.03 | -0.0049 | 0.093 | 0.17 | -0.0373 | **0.001** | **0.48** | **-0.0768** |
| Longitude (dd) | -120.107 | -114.343 | 0.620 | 0.02 | 0.0016 | 0.539 | 0.02 | -0.0064 | 0.147 | 0.13 | -0.0178 |
| Elevation (m) | 1981 | 2454 | 0.187 | 0.11 | -0.0001 | 0.118 | 0.15 | -0.0002 | 0.219 | 0.09 | -0.0002 |
| MAT (˚C) | -3.4 | 1.1 | 0.352 | 0.05 | 0.0038 | 0.090 | 0.17 | 0.0226 | **<0.001** | **0.58** | **0.0504** |
| MWMT (˚C) | 8.1 | 12.3 | 0.218 | 0.09 | 0.0051 | 0.348 | 0.06 | 0.0131 | 0.039 | 0.24 | 0.0329 |
| MCMT (˚C) | -14.6 | -7.4 | 0.771 | 0.01 | 0.0007 | 0.062 | 0.20 | 0.0151 | **<0.001** | **0.69** | **0.0337** |
| TD (˚C) | 17.1 | 24.1 | 0.608 | 0.02 | 0.0015 | 0.153 | 0.12 | -0.0135 | 0.007 | 0.37 | -0.0284 |
| Tmin_at (˚C) | -7.1 | -1.7 | 0.571 | 0.02 | 0.0018 | 0.064 | 0.20 | 0.0192 | **<0.001** | **0.66** | **0.0423** |
| MAP (mm) | 773 | 1853 | 0.869 | 0.00 | 0.0000 | 0.101 | 0.16 | 0.0001 | 0.821 | 0.00 | 0.0000 |
| MSP (mm) | 242 | 681 | 0.929 | 0.00 | 0.0000 | 0.518 | 0.03 | -0.0001 | 0.020 | 0.30 | -0.0004 |
| AHM (˚C/µm) | 4.5 | 13.3 | 0.860 | 0.00 | 0.0004 | 0.721 | 0.01 | -0.0027 | 0.161 | 0.12 | 0.0123 |
| SHM (˚C/µm) | 13 | 40.2 | 0.843 | 0.00 | 0.0001 | 0.498 | 0.03 | 0.0015 | 0.009 | 0.36 | 0.0064 |
| DD_0 (days) | 1165 | 2140 | 0.481 | 0.03 | 0.0000 | 0.077 | 0.18 | -0.0001 | **<0.001** | **0.65** | **-0.0002** |
| DD_5 (days) | 300 | 712 | 0.231 | 0.09 | 0.0000 | 0.253 | 0.08 | 0.0002 | 0.023 | 0.28 | 0.0003 |
| NFFD (days) | 91 | 150 | 0.557 | 0.02 | 0.0002 | 0.068 | 0.19 | 0.0017 | **0.001** | **0.53** | **0.0035** |
| FFP (days) | 51 | 105 | 0.862 | 0.00 | 0.0001 | 0.026 | 0.27 | 0.0021 | **<0.001** | **0.56** | **0.0036** |
| bFFP (date) | 152 | 181 | 0.944 | 0.00 | 0.0000 | 0.036 | 0.25 | -0.0038 | 0.009 | 0.35 | -0.0055 |
| eFFP (date) | 231 | 258 | 0.620 | 0.02 | 0.0003 | 0.038 | 0.24 | 0.0037 | **<0.001** | **0.67** | **0.0074** |
| PAS (mm) | 503 | 1238 | 0.996 | 0.00 | 0.0000 | 0.052 | 0.22 | 0.0002 | 0.650 | 0.01 | 0.0000 |
| EMT (˚C) | -50 | -37.4 | 0.816 | 0.00 | 0.0003 | 0.075 | 0.18 | 0.0078 | **<0.001** | **0.62** | **0.0173** |
| EXT (˚C) | 25.5 | 29.5 | 0.151 | 0.12 | 0.0057 | 0.569 | 0.02 | 0.0078 | 0.163 | 0.12 | 0.0224 |
| Eref (mm) | 287 | 421 | 0.118 | 0.15 | 0.0002 | 0.945 | 0.00 | 0.0000 | 0.482 | 0.03 | 0.0004 |
| CMD (mm) | 0 | 80 | 0.974 | 0.00 | 0.0000 | 0.698 | 0.01 | 0.0003 | 0.055 | 0.21 | 0.0015 |
